# Four-Step Synthesis of 3-Allyl-2-(allyloxy)-5-bromoaniline from 2-Allylphenol

**DOI:** 10.3390/m1773

**Published:** 2024-02-07

**Authors:** Enrique B. Aparicio, Stephen R. Isbel, Alejandro Bugarin

**Affiliations:** Department of Chemistry and Physics, Florida Gulf Coast University, 10501 FGCU Boulevard South, Fort Myers, FL 33965, USA

**Keywords:** 2-allylphenol, nitration, allylic ether, aniline

## Abstract

This communication reports a four-step protocol to produce 3-allyl-2-(allyloxy)-5-bromoaniline **5** from commercially available 2-allylphenol. The synthetic steps used were nitration, selective bromination, allylation, and reduction of the nitro group.

## 1. Introduction

Numerous natural products and biologically active compounds incorporate nitrogen within their structures, rendering nitrogen-containing molecules valuable for the advancement of pharmaceuticals and other biologically pertinent substances [1–3]. Anilines and aminophenols are among those types of nitrogen-containing and versatile building blocks [4], which play a key role in diverse syntheses, especially in medicinal chemistry [5–7] and the dye industry [8]. Hence, there is significant interest in developing new synthetic routes toward functionalized aniline derivatives. A research interest in our group is centered on the mild C-H activation of miscellaneous allylbenzenes (including anilines). For instance, we have developed transition metal-free protocols for the synthesis of (*E*)-allylic compounds [9] and α-alkyl styrenes [10] from terminal alkenes, sometimes using additives or heat to improve yields. While mechanistic studies have helped to better understand the electronic effects in these transformations using allylbenzenes [11], it is still necessary to perform a direct comparison between allylbenzenes and allyl ethers. As such, the title compound was designed, synthesized, and characterized as described below.

## 2. Results and Discussion

**Scheme 1.**
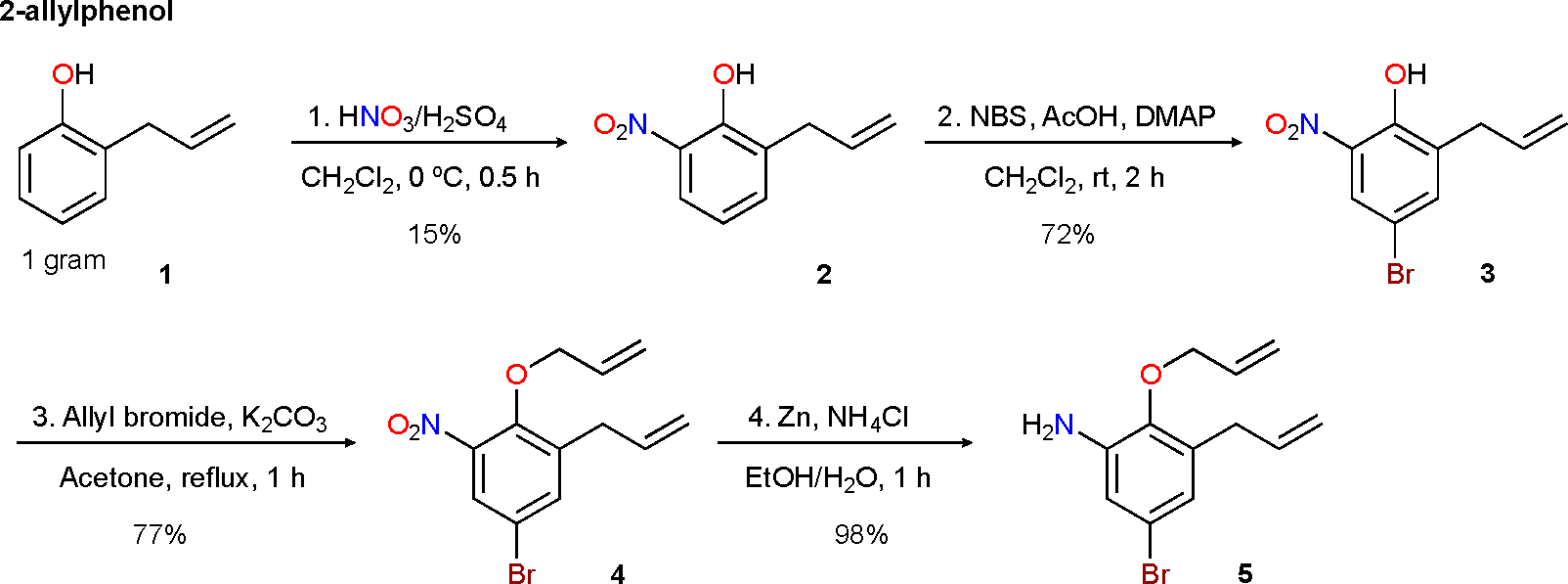
Four-step synthesis of 3-allyl-2-(allyloxy)-5-bromoaniline **5**.

As mentioned above, we embarked on the design of a benzene derivative bearing both allyl moieties (allylbenzene and allyl ether). We also wanted the new compound to be electron-rich (therefore, an amino group was also envisioned) and have a larger mass (bromo was added). With these requirements in mind, the synthesis started. Although the precedent literature uses Claisen rearrangement to install allyl groups into the benzene ring of phenols [12], we decided to take an alternative route. The commercially available 2-allylphenol **1** was used as it already has the allyl group (Scheme 1). First, 1 g of 2-allylphenol **1** was subjected to nitration conditions using a sulfonitric mixture (HNO_3_/H_2_SO_4_) and reacted for 30 min. at 0 °C. Analysis of the reaction mixture showed two products: the expected 2-allyl-6-nitrophenol **2** in 15% yield and 2-allyl-4-nitrophenol (not shown) in 15% yield [13]. Then, 2-allyl-6-nitrophenol **2** was selectively brominated using *N*-bromosuccinimide (NBS), which afforded 2-allyl-4-bromo-6-nitrophenol **3** in 72% yield [14]. The phenol group on **3** was alkylated under standard conditions using allyl bromide [12], producing 1-allyl-2-(allyloxy)-5-bromo-3-nitrobenzene **4** in 77% yield. Lastly, the nitro group in intermediate **4** was reduced using Zn and NH_4_Cl [15], after only 1 h of reaction, which delivered the expected new product 3-allyl-2-(allyloxy)-5-bromoaniline **5** in 98% isolated yield (Scheme 1). The spectra of compounds **2–4** can be found in the Supplementary Materials.

## 3. Materials and Methods

### 3.1. General Information

All reactions were carried out in the air in oven-dried glassware with magnetic stirring at room temperature. 2-Allylphenol was purchased from Millipore Sigma (St. Louis, MO, USA) and used as received. All reagents and solvents were purchased from Fisher (Hampton, NH, USA) and used as received. The purification of reaction products was carried out by flash column chromatography using silica gel 60 (230–400 mesh). TLC visualization was accompanied by UV light. Concentration in vacuo refers to the removal of volatile solvent using a rotary evaporator attached to a dry diaphragm pump (10–15 mm Hg), followed by pumping to a constant weight with an oil pump (<300 mTorr).

^1^H NMR spectra were recorded at 400 MHZ (Jeol, Akishima, Tokyo, Japan) and are reported relative to CDCl_3_ (δ = 7.26). ^1^H NMR coupling constants (*J*) are reported in Hertz (Hz), and multiplicities are indicated as follows: s (singlet), d (doublet), t (triplet), and m (multiplet). Proton-decoupled ^13^C NMR spectra were recorded at 100 MHz and reported relative to CDCl_3_ (δ = 77). IR experiments were recorded with neat samples on a Jasco FT/IR-4700 (Easton, MD, USA) fitted with a diamond ATR sample plate. GCMS data was recorded on a Shimadzu GC-2010 plus (Kyoto, Kyoto, Japan) system (GCMS-QP2010 SE). Elemental analysis measurements were recorded on a FlashSmart elemental analyzer (Thermo Fisher Scientific, Waltham, MA, USA).

### 3.2. Synthesis of 2-Allyl-6-nitrophenol 2

Compound **2** was synthesized following a modified procedure described in the literature [13]: To a 20 mL scintillation vial, equipped with a stir bar, 2-allylphenol (7.45 mmol, 1.0 equiv., 1 g) and dichloromethane (7 mL) were added, then chilled to 0 °C (ice bath). After being cooled, 2 mL of sulfonitric mixture (prepared with 2 mL of concentrated nitric acid, 6 mL of sulfuric acid, and 2 mL of water) was added dropwise. Then, the mixture was allowed to warm to room temperature and stirred for 30 min, followed by the addition of water (10 mL). This crude mixture was directly added to a silica gel column for purification. Purification by flash chromatography [silica gel, hexanes/ethyl acetate (99:1)] provided the pure product **2** as a bright yellow oil (193.2 mg, 15% yield). Rf = 0.69 [hexanes/ethyl acetate (4:1)]. Note: 2-allyl-4-nitrophenol (not shown) was observed as red oil [191 mg, 15% yield, Rf = 0.24 [hexanes/ethyl acetate (4:1)].

^1^H NMR (400 MHz, CDCl_3_) δ 10.96 (s, 1H, OH), 8.00 (1H, d, *J* = 8.6 Hz, H-5), 7.47 (1H, d, *J* = 7.3 Hz, H-3), 6.93 (1H, t, *J* = 8.0 Hz, H-4), 6.04–5.94 (1H, m, H-2′), 5.15–5.09 (2H, m, H-3′), 3.49 (2H, d, *J* = 6.5 Hz, H-1′). ^13^C NMR (101 MHz, CDCl3) δ 153.27, 137.51, 135.09, 133.57, 131.35, 123.1, 119.48, 116.82, 33.62. IR (neat, cm^−1^): ν 3205 (OH); 3085 (=C-H); 2981 (C-H); 1608 (C=C); 1539 (-NO_2_); 1450 (C=C); 1327 (N=O); 1249 (C-O-C). LRMS (EI) Calcd for C_9_H_9_NO_3_ [M], 179.05. Found: 179[M]. The analytic data are in accordance with the data reported in the literature [13].

### 3.3. Synthesis of 2-Allyl-4-bromo-6-nitrophenol 3

Compound **3** was synthesized by a modification of a procedure described in the literature [14]: To a 20 mL scintillation vial, equipped with a stir bar, 2-allyl-6-nitrophenol **2** (0.70 mmol, 1.0 equiv., 123.8 mg), dichloromethane (5 mL), glacial acetic acid (0.5 mL), 4-dimethylaminopyridine (0.07 mmol, 0.1 equiv., 8.6 mg), and *N*-bromosuccinimide (0.71 mmol, 1.01 equiv., 126.4 mg) were added at room temperature. Then, the mixture was stirred at room temperature for 2 h. This crude mixture was directly added to a silica gel column for purification. Purification by flash chromatography [silica gel, hexanes (100%)] provided the pure product **3** as bright yellow oil (134.1 mg, 72% yield). Rf = 0.88 [hexanes/ethyl acetate (4:1)].

^1^H NMR (400 MHz, CDCl_3_) δ 10.87 (s, 1H, OH), 8.14 (1H, d, *J* = 2.2 Hz, H-5), 7.56 (1H, d, *J* = 2.4 Hz, H-3), 6.08–5.9 (1H, m, H-2′), 5.2–5.13 (2H, m, H-3′), 3.47 (2H, d, *J* = 5.9 Hz, H-1′). ^13^C NMR (101 MHz, CDCl_3_) δ 152.31, 139.94, 134.10, 133.83, 133.70, 125.17, 117.75, 111.23, 33.40. IR (neat, cm^−1^): ν 3208 (OH); 3089 (=C-H); 2919 (C-H); 1604 (C=C); 1531 (−NO_2_); 1450 (C=C); 1319 (N=O); 1238 (C-O-C); 663 (C-Br). LRMS (EI) Calcd for C_9_H_8_BrNO_3_ [M], 256.96. Found: 257[M], 259[M + 2]. The analytic data is in accordance with the data reported in the literature [15].

### 3.4. Synthesis of 1-Allyl-2-(allyloxy)-5-bromo-3-nitrobenzene 4

Compound **4** was synthesized by a modification of procedures described in the literature [12,15–17]: To a 20 mL scintillation vial, equipped with a stir bar, was added 2-allyl-4-bromo-6-nitrophenol **3** (0.52 mmol, 1.0 equiv., 134.1 mg), potassium carbonate (0.78 mmol, 1.5 equiv., 107.8 mg), acetone (5 mL), and allyl bromide (0.78 mmol, 1.5 equiv., 94.4 mg, 67 μL), at room temperature. Then, the mixture was heated to reflux and stirred for 1 h. This crude mixture was directly added to a silica gel-column chromatography for purification. Purification by flash chromatography [silica gel, hexanes/ethyl acetate (99:1)] provided the pure product **4** as a light orange oil (119.7 mg, 77% yield). Rf = 0.56 [hexanes/ethyl acetate (4:1)].

^1^H NMR (400 MHz, CDCl_3_) δ 7.81 (1H, d, *J* = 2.2 Hz, H-5), 7.55 (1H, d, *J* = 2.4 Hz, H-3), 6.08–5.91 (2H, m), 5.41–5.10 (4H, m), 4.47 (2H, d, *J* = 6.1 Hz, H-1″), 3.45 (2H, d, *J* = 6.6 Hz, H-1′). ^13^C NMR (101 MHz, CDCl_3_) δ 149.22, 144.75, 138.81, 137.59, 134.79, 132.27, 126.09, 119.24, 117.97, 116.15, 76.17, 33.5. IR (neat, cm^−1^): ν 3081 (=C-H); 2923 (C-H); 1643 (C=C); 1531 (−NO_2_); 1461 (C=C); 1349 (N=O); 1253 (C-O-C); 705 (C-Br). LRMS (EI) Calcd for C_12_H_12_BrNO_3_ [M], 297.00. Found: 297[M], 299[M + 2]. The analytic data is in accordance with the reported in the literature [15].

### 3.5. Synthesis of 3-Allyl-2-(allyloxy)-5-bromoaniline 5

The new compound 3-allyl-2-(allyloxy)-5-bromoaniline **5** was synthesized by a modification of procedures described in the literature [12,15]: To a 20 mL scintillation vial, equipped with a stir bar, the pure 1-allyl-2-(allyloxy)-5-bromo-3-nitrobenzene **4** (0.1 mmol, 1.0 equiv., 26.7 mg), EtOH (1 mL), ammonium chloride (1.0 mmol, 10.0 equiv., 57.9 mg), zinc (1.0 mmol, 10.0 equiv., 64.7 mg), and water (0.5 mL) were added sequentially. The reaction was heated to reflux, immediately allowed to cool to rt, and stirred for 1 h. Then, the reaction mixture was filtered using a syringe filter (PVDF-L membrane filter, 0.45 μm) to eliminate the solids. The volatiles were removed under reduced pressure. Purification by flash chromatography [silica gel, hexanes/EtOAc (90:10)] provided the pure product **5** as a light-yellow oil (23.6 mg, 98% yield). Rf = 0.45 [hexanes/ethyl acetate (4:1)].

^1^H NMR (400 MHz, CDCl_3_) δ 6.76 (1H, d, *J* = 2.4 Hz, H-5), 6.69 (1H, d, *J* = 2.4 Hz, H-3), 6.13–6.03 (1H, m), 5.97–5.87 (1H, m), 5.43 (1H, dq, *J* = 17.2, 1.7 Hz), 5.28 (1H, dq, *J* = 10.5, 1.4 Hz), 5.14–5.04 (2H, m), 4.32 (2H, dt, *J* = 5.5, 1.4 Hz, H-1″), 3.82 (2H, s, NH_2_), 3.34 (2H, dt, *J* = 6.6, 1.5 Hz, H-1′). ^13^C NMR (101 MHz, CDCl_3_) δ 143.17, 141.48, 136.38, 135.12, 133.65, 122.19, 117.65, 117.27, 116.57, 116.36, 72.98, 33.68. IR (neat, cm^−1^): ν 3467 (NH); 3374 (NH′); 3077 (=C-H); 2919 (C-H); 1608 (C=C); 1477 (C=C); 1195 (C-O-C); 674 (C-Br). LRMS (EI) Calcd for C_12_H_14_BrNO [M], 267.02. Found: 267[M], 269[M + 2]. Elemental analysis calculated (%) for C_12_H_14_BrNO: C 53.75, H 5.26, N 5.22. Found: C 53.71, H 5.24, N 5.18.

## 4. Conclusions

In summary, this communication described a four-step approach for the synthesis of 3-allyl-2-(allyloxy)-5-bromoaniline 5 from 2-allylphenol. The synthetic method is simple. Besides the first reaction (15%), the yields achieved in our study are good to excellent (72–98%). All the steps provide pure products after isolation using silica gel column chromatography.

## Supplementary Material

Supplementary Material

## Data Availability

The data are contained within the article.
