# Supplementary material for: Four-Step Synthesis of 3-Allyl-2-(allyloxy)-5-bromoaniline from 2-Allylphenol
Source: Molbank. Author manuscript; Available in PMC 2025 Feb 27. (PMC11867607; doi:10.3390/m1773)
Supplement: Supplementary Material [file NIHMS2014591-supplement-Supplementary_Material.docx]

**Four-step Synthesis of 3-Allyl-2-(allyloxy)-5-bromoaniline from 2-Allylphenol**

Enrique B. Aparicio, Stephen R. Isbel, and Alejandro Bugarin*

* Department of Chemistry and Physics, Florida Gulf Coast University,

10501 FGCU Boulevard South, Fort Myers, FL 33965

[abugarin@fgcu.edu](mailto:abugarin@fgcu.edu)

**Table of Contents**

^1^H and ^13^C NMR Spectra of Compound **2** ………….……….………………… S-2

GC-Mass Spectrum of Compound **2** …………………….……….……………… S-3

IR Spectrum of Compound **2** ………….……………….…….………………… S-4

^1^H and ^13^C NMR Spectra of Compound **3** ………….……….………………… S-5

GC-Mass Spectrum of Compound **3** …………………….……….……………… S-6

IR Spectrum of Compound **3** ………….……………….…….………………… S-7

^1^H and ^13^C NMR Spectra of Compound **4** ………….……….………………… S-8

GC-Mass Spectrum of Compound **4** …………………….……….……………… S-9

IR Spectrum of Compound **4** ………….……………….…….………………… S-10

^1^H and ^13^C NMR Spectra of Compound **5** ………….……….………………… S-11

GC-Mass Spectrum of Compound **5** …………………….……….……………… S-12

IR Spectrum of Compound **5** ………….……………….…….………………… S-13

Elemental Analysis Chromatogram of Compound **5**………….…………. …… S-14

**Figure S1.** ^1^H NMR (400 MHz, CDCl_3_)

**2**

**Figure S2.** ^13^C NMR (101 MHz, CDCl_3_)

**2**

**Figure S3.** Mass Spectrum of Compound **2**


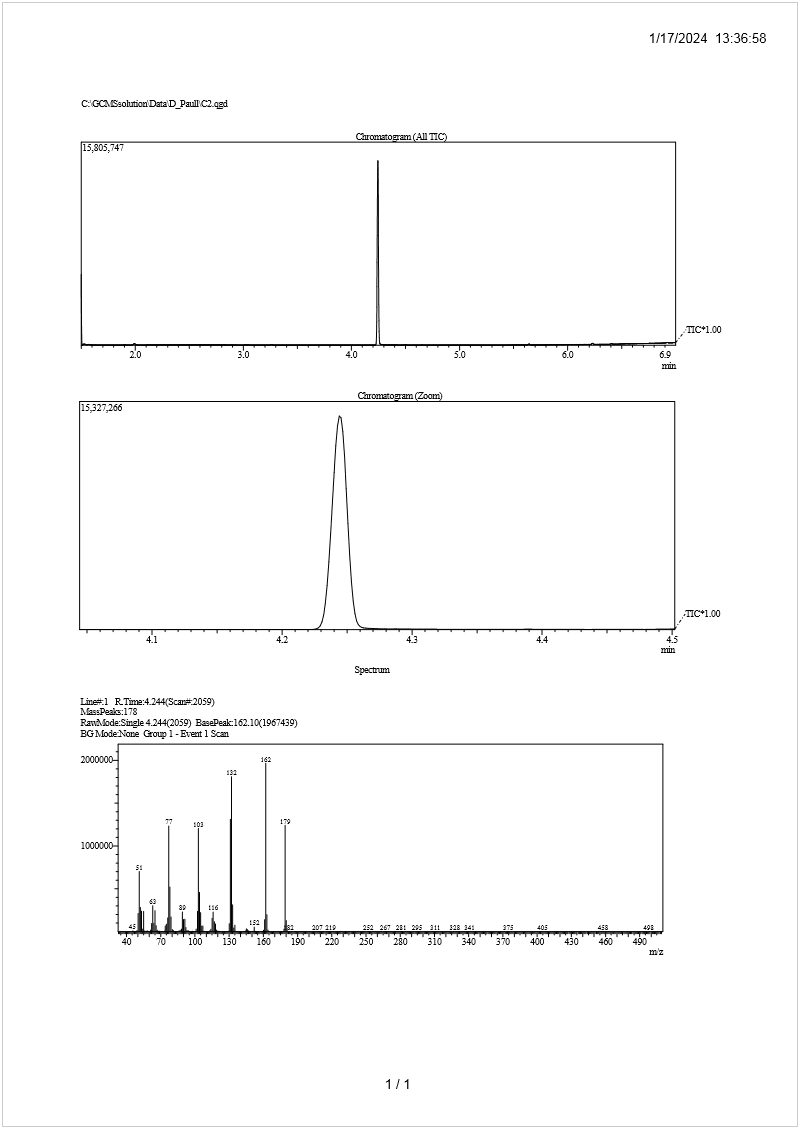


**2,** MW = 179

**Figure S4.** IR Spectrum of Compound **2**

**2**

**Figure S5.** ^1^H NMR (400 MHz, CDCl_3_)

**3**

**Figure S6.** ^13^C NMR (101 MHz, CDCl_3_)

**3**

**Figure S7.** Mass Spectrum of Compound **3**


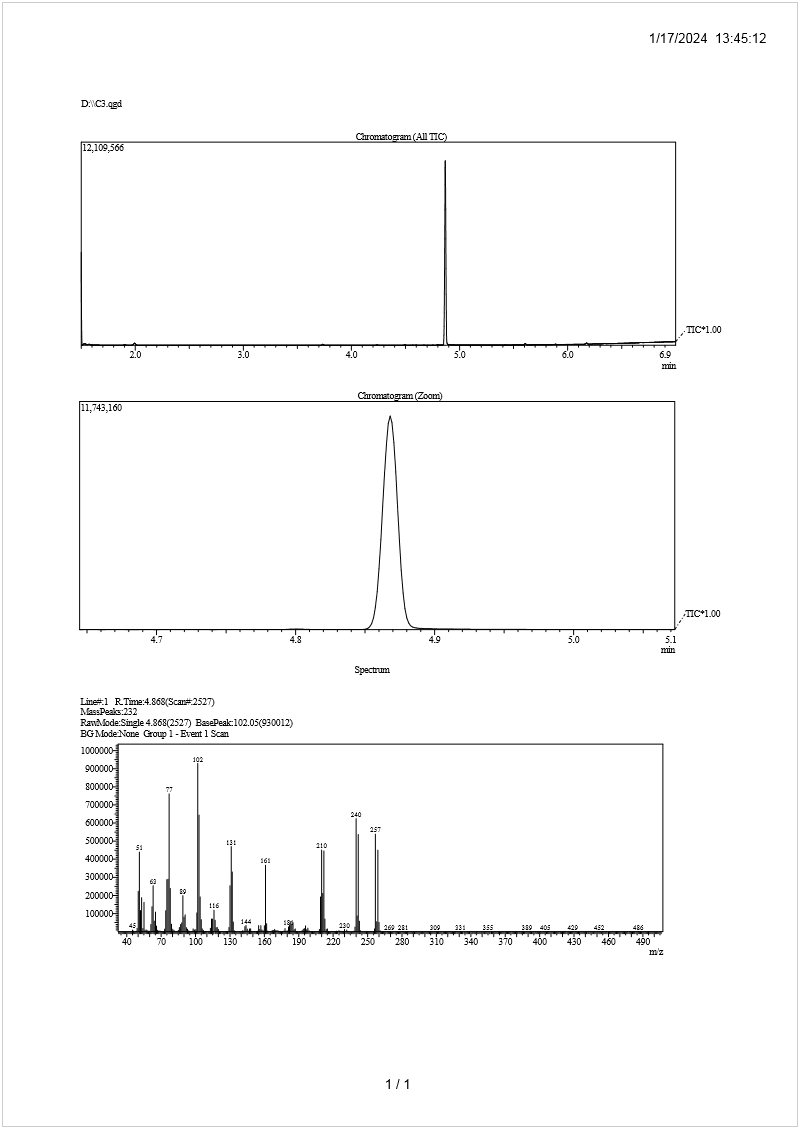


**3**, MW = 257

**Figure S8.** IR Spectrum of Compound **3**

**3**

**Figure S9.** ^1^H NMR (400 MHz, CDCl_3_)

**4**

**Figure S10.** ^13^C NMR (101 MHz, CDCl_3_)

**4**

**Figure S11.** Mass Spectrum of Compound **4**


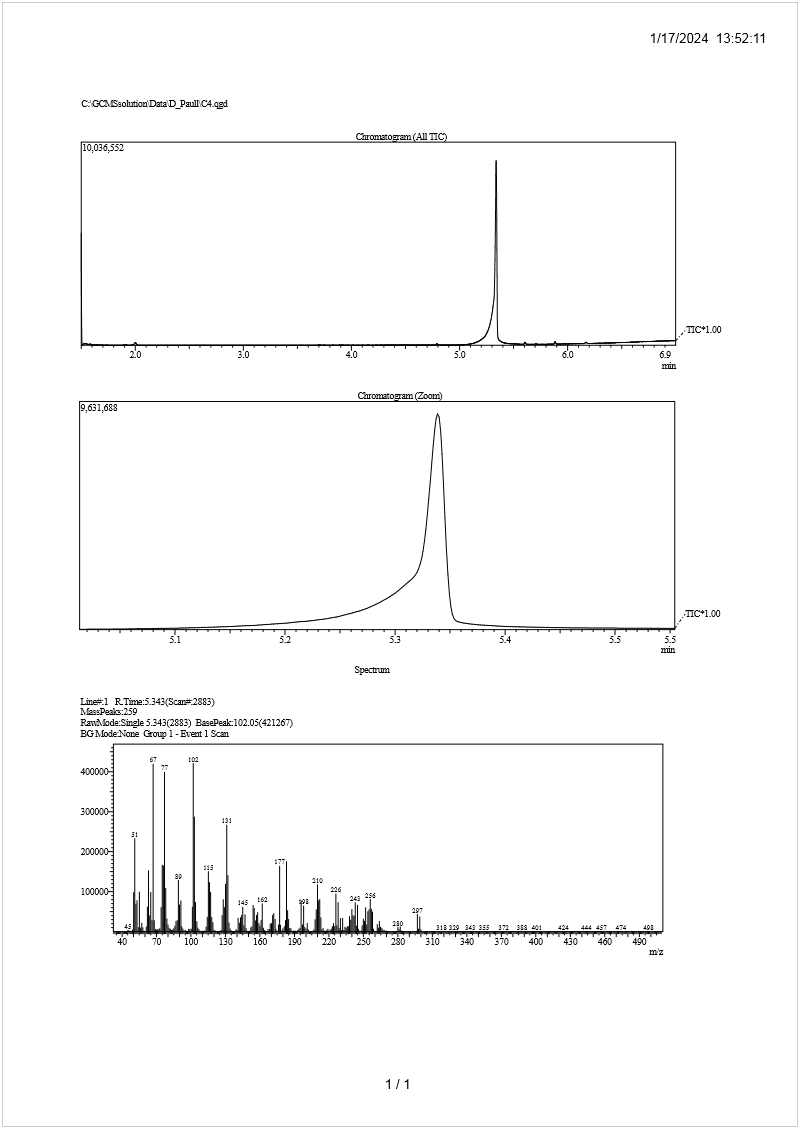


**4**, MW =297

**Figure S12.** IR Spectrum of Compound **4**

**4**

**Figure S13.** ^1^H NMR (400 MHz, CDCl_3_)

**5**

**Figure S14.** ^13^C NMR (101 MHz, CDCl_3_)

**5**

**Figure S15.** Mass Spectrum of Compound **5**


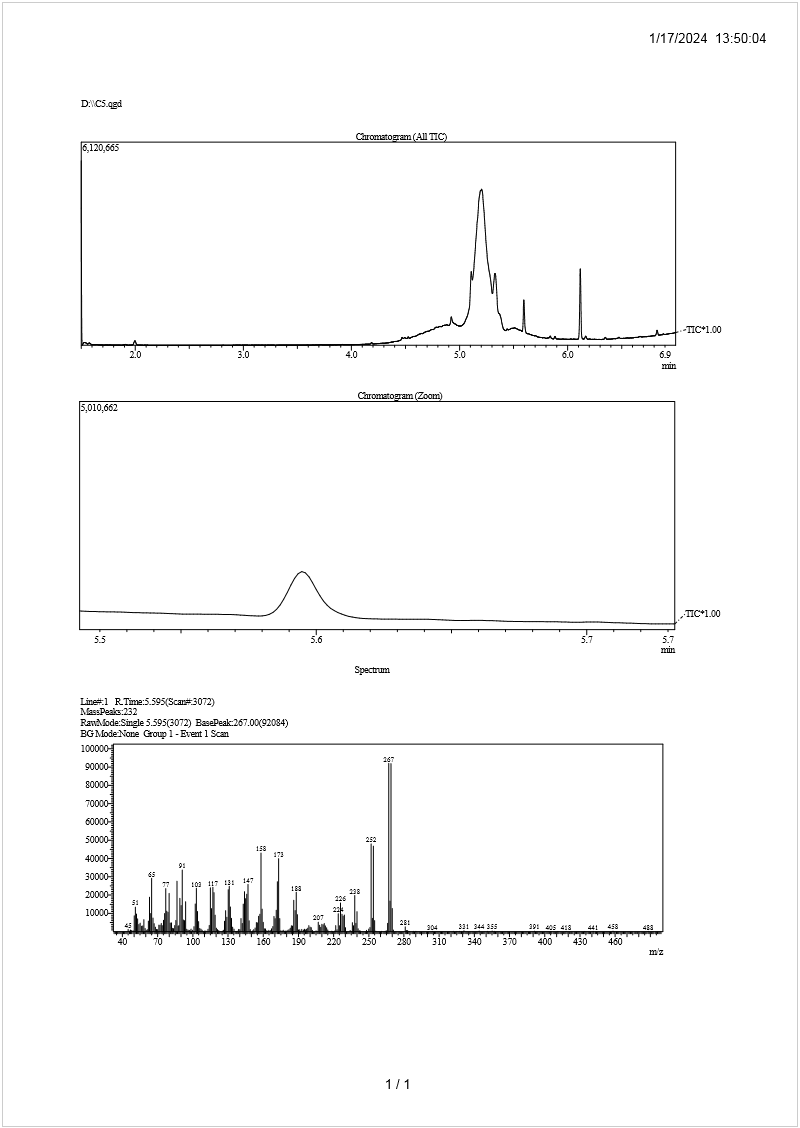


**5**, MW =267

**Figure S16.** IR Spectrum of Compound **5**

**5**

**Figure S17.** Elemental Analysis Chromatogram of Compound **5**

**5**

|  |  | Nitrogen | Carbon | Hydrogen | Sulphur |
| --- | --- | --- | --- | --- | --- |
| 1 | bypass | 0 | 0 | 0 | 0 |
| 2 | blank | 0 | 0 | 0 | 0 |
| 3 | Std: BBOT | 6.51 | 72.53 | 6.09 | 0 |
| 5 | Compound **5** | 5.18 | 53.71 | 5.24 | 0 |
